# Global disease burden of colorectal cancer attributable to high BMI has more than doubled over the past 30 years

**DOI:** 10.3389/fonc.2025.1580458

**Published:** 2025-05-19

**Authors:** Jiamin Zeng, Wenjie Li, Zhiming Wu

**Affiliations:** ^1^ Department of Urology, Sun Yat-sen University Cancer Center, Guangzhou, China; ^2^ State Key Laboratory of Oncology in Southern China, Guangzhou, China; ^3^ Collaborative Innovation Center of Cancer Medicine, Guangzhou, China; ^4^ State Key Laboratory of Oncology in South China, Guangdong Provincial Clinical Research Center for Cancer, Guangzhou, China; ^5^ Department of Radiation Oncology, Nanfang Hospital, Southern Medical University, Guangzhou, China

**Keywords:** colorectal cancer, high body mass index, global burden, prevention, public health

## Abstract

**Background:**

Colorectal cancer (CRC) significantly contributes to global cancer-related mortality and morbidity, with a high body mass index (BMI) being a key modifiable risk factor. Understanding the evolving burden of CRC attributable to high BMI is essential for informing public health strategies and meeting global noncommunicable disease targets.

**Methods:**

Using data from the Global Burden of Disease Study 2021, we examined the age-, sex-, and location-specific CRC burden attributable to obesity. Trends in age-standardized death rates (ASDR) and disability-adjusted life-years (DALYs) were assessed using the estimated annual percentage change (EAPC).

**Results:**

Between 1990 and 2021, CRC deaths attributable to obesity increased from 41,535.8 (95% UI 17,665.6–67,379) to 99,268.0 (95% UI 42,956.3–157,948.8), while DALYs increased from 15,042.1 (95% UI 4,297.8–16,319.7) to 64,664.2 (95% UI 102,159.3–375,234.0). High-SDI regions showed declining ASDR (EAPC = −0.64, 95% UI −0.69 to −0.59) and DALY rates (EAPC = −0.48, 95% UI −0.52 to −0.43) but retained the highest absolute burden. In contrast, the middle- and low-SDI regions exhibited alarming increases in both ASDR and DALY rates, with EAPCs exceeding 2.0. East Asia reported the highest absolute mortality and DALY burden, whereas Australasia showed the lowest burden and declining trends. Inequality in the CRC burden widened substantially between the high- and low-SDI regions during the study period.

**Conclusion:**

The global burden of CRC attributable to high BMI doubled from 1990 to 2021, with increasing disparities across SDI regions, especially in the low- and middle-SDI areas. Urgent strategies focusing on obesity prevention, early detection, and equitable care are essential to reduce this burden and achieve Sustainable Development Goals by 2030.

## Highlights

- Over the last 30 years, the burden of high BMI-related CRC has doubled, with predictions suggesting an annual increase of more than 150,000 cases by 2040.
- High SDI regions maintain the highest global CRC burden despite declining trends, whereas the middle and low SDI regions face rapid increases.
- East Asia leads to absolute CRC mortality and DALYs related to high BMI driven by rapid economic development and lifestyle shifts.
- Low middle- or low-income regions, while reporting the lowest absolute burden, showed worrisome upward trends.
- Significant increases in absolute inequality highlight a growing gap in the CRC burden between high and low SDI regions.

## Introduction

Colorectal cancer (CRC) is the third most common cause of cancer-related mortality and the second leading cause of disability-adjusted life years (DALYs) worldwide (1). Sporadic cases constitute approximately 70%–75% of all CRC cases and are strongly associated with modifiable risk factors, such as obesity (2, 3). For instance, a meta-analysis reported that individuals with obesity have a 30% higher risk of developing early onset CRC than those with a normal BMI (4). The increasing prevalence of obesity is driving an increase in CRC incidence, particularly in regions experiencing upward trends in body mass index (BMI) (5–7). Furthermore, high BMI has been shown to be robustly correlated with an increased risk of early onset CRC. These associations remained significant even after controlling for several potential confounding factors (8). The concerning evolution underscores the critical necessity for targeted interventions aimed at modifying diet and lifestyle to mitigate risk factors, such as high BMI, since the effective prevention of CRC and implementation of established screening protocols for early detection have been shown to substantially decrease mortality rates (9).

Despite growing evidence linking obesity to CRC, comprehensive analyses of the global burden of CRC attributable to high BMI remain limited. While previous studies have highlighted the rising incidence of CRC associated with obesity, they often focus on specific regions or populations, lack detailed temporal trends across diverse sociodemographic contexts, or fail to quantify the health loss in terms of both mortality and disability (2, 3, 5, 6). Moreover, there is a paucity of research examining how the CRC burden attributable to high BMI varies across countries with different levels of development, as measured by the Sociodemographic Index (SDI), and how these disparities evolve over time. This knowledge gap hinders the development of targeted public health strategies to address the global CRC burden, particularly in the context of achieving the United Nations Sustainable Development Goal (SDG) target 3.4, which aims to reduce premature mortality from non-communicable diseases by one-third by 2030. To address this gap, our study systematically evaluated the global, regional, and national burden of CRC attributable to high BMI in 204 countries and territories from 1990 to 2021, offering valuable insights into the epidemiological patterns of CRC over recent decades. We examined the age-sex-location-specific burden of CRC attributable to high BMI using age-standardized death rates (ASDR) and disability-adjusted life years (DALYs) in relation to countries’ development status, as measured by the SDI, with trends assessed using estimated annual percentage change (EAPC) estimates from the Global Burden of Diseases Study (GBD) 2021.

## Results

### Overview of the global burden, age, and sex patterns

**Figure 1:**
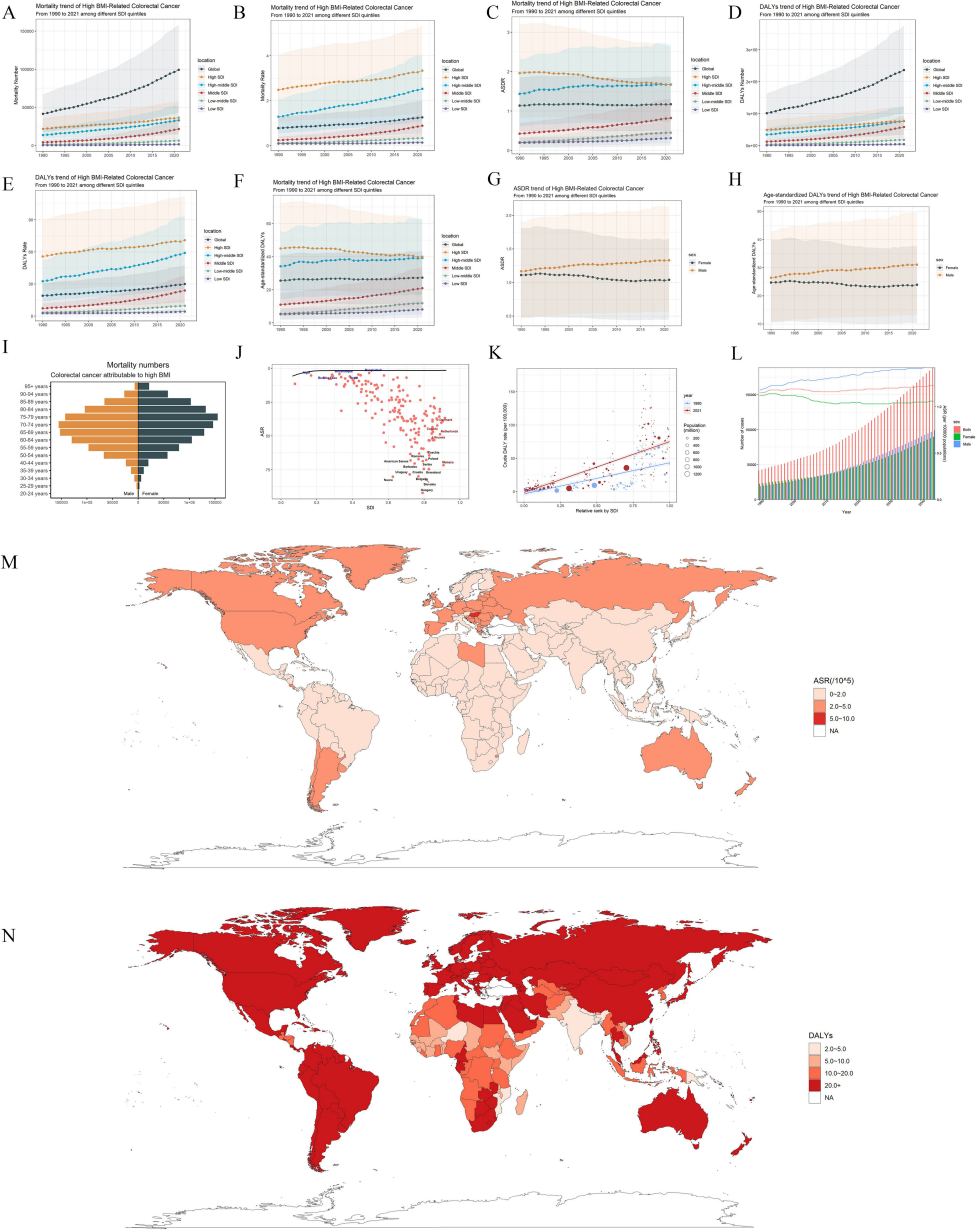
Global trends and regional disparities in colorectal cancer (CRC) burden attributable to high BMI from 1990 to 2021. **(A–F)** Trends in mortality and disability-adjusted life-years (DALYs) attributable to high BMI-related CRC across sociodemographic index (SDI) quintiles from 1990 to 2021. Panels **(A)**, **(B)**, and **(C)** show mortality number, all-age mortality rate and age-standardized death rate trends, while panels **(D)**, **(E)**, and **(F)** show DALYs number, all-age DALYs rate, and age-standardized DALY rate trends. −0.65 (−0.71–−0.59). **(I)** Global age distribution of age-standardized mortality rates (ASDR) for CRC attributable to high BMI in 2021. **(J)** Frontier analysis with the black line representing the defined frontier. Black dots correspond to the 14 countries exhibiting the greatest effective difference. Blue dots highlight the top five low-SDI countries with the most pronounced effective difference, while red dots indicate high-SDI countries and territories demonstrating the largest effective difference. **(K)** Health inequality analysis. **(L)** Projected trends in CRC cases attributable to high BMI by sex and year. **(M, N)** The age-standardized death rates of colorectal cancer attributable to obesity for 204 countries and territories. **(M)** age-standardized death rate; **(N)** disability-adjusted life-years.

Globally, the burden of CRC attributable to a high BMI has consistently increased between 1990 and 2021. In 1990, CRC deaths attributable to high BMI were estimated to be 41,535.8 (95% UI 17,665.6–67,379), which more than doubled by 2021, reaching 99,268.0 (95% UI 42,956.3–157,948.8) (**Supplementary Table 1**, **Figures 1A, B**). This increase was accompanied by a modest rise in the global ASDR, from 1.1 (95% UI 0.5–1.9) per 100,000 in 1990 to 1.2 (95% UI 0.5–1.9) per 100,000 in 2021 (**Supplementary Table 1**, **Figure 1C**). Similarly, the global burden of DALYs attributable to high BMI increased dramatically, from 15,042.1 (95% UI 4,297.8–16,319.7) in 1990 to 64,664.2 (95% UI 102,159.3–375,234.0) in 2021 (**Table 1**, **Figures 1D, E**). The age-standardized DALY rate also showed a modest increase during this period, from 25.5 (95% UI 10.8–41.2) per 100,000 in 1990 to 27.3 (95% UI 11.8–43.4) in 2021 (**Table 1**, **Figure 1F**). Furthermore, males consistently demonstrated higher levels of ASDR and DALYs than females, who exhibited a modest declining trend over three decades (**Figures 1G, H**). The incidence count followed a bell-shaped distribution, with a peak in individuals aged 65–79 years for both males and females (**Figure 1I**).

**Table 1:** The mortality cases and ASDR as well as DALYs of colorectal cancer attributed to high body mass index in 1990 and 2021.

Location	ASDR					DALYs				
	1990		2021		1990–2021 EAPC (95% CI)	1990		2021		1990–2021 EAPC (95% CI)
	Number (95% UI)	ASDR (95% UI)	Number (95% UI)	ASDR (95% UI)		Number (95% UI)	DALYs (95% UI)	Number (95% UI)	DALYs (95% UI)	
Global	41,535.8 (17,665.6–67379)	1.1 (0.5–1.9)	99,268 (42,956.3–157,948.8)	1.2 (0.5–1.9)	0 (−0.04–0.04)	1,015,042.1 (429,787.2–1,631,973.8)	25.5 (10.8–41.2)	2,364,664.2 (1,021,593.6–3,752,340.4)	27.3 (11.8–43.4)	0.12 (0.08–0.16)
SDI										
High SDI	21,852.2 (9,256.4–35,533.2)	2 (0.8–3.2)	36,529.9 (15,670.3–58,138.7)	1.7 (0.7–2.7)	−0.64 (−0.69–−0.59)	490,182.6 (210,224.6–788,176.6)	44.9 (19.3–72.2)	775,808.6 (337,834.3–1,225,933.9)	40 (17.5–62.9)	−0.48 (−0.52–−0.43)
High-middle SDI	13,679.9 (5,864.9–22,167.7)	1.4 (0.6–2.3)	32,966.4 (14,288.4–52,368.1)	1.7 (0.7–2.7)	0.4 (0.32–0.48)	346,229.2 (147,488.9–559,452.8)	34.2 (14.6–55.3)	769,289.9 (332,395–1,220,597.7)	39.2 (16.9–62.3)	0.31 (0.24–0.37)
Middle SDI	4,237.3 (1,566.5–6,974.5)	0.4 (0.2–0.7)	21,653.8 (9,249.9–34,503.1)	0.8 (0.4–1.3)	2.13 (2.11–2.16)	125,727.8 (46,880.4–206,617.4)	11 (4.1–18.1)	584,511.4 (248,469.1–929,607.8)	21 (8.9–33.5)	2.1 (2.07–2.14)
Low-middle SDI	1,244.5 (472.1–1,981.8)	0.2 (0.1–0.3)	6,391.2 (2,689.5–10,103.4)	0.5 (0.2–0.7)	2.72 (2.65–2.79)	37,581.2 (14,269–60,129.2)	5.5 (2.1–8.8)	184,447.8 (77,501.9–290,094.4)	11.9 (5–18.7)	2.69 (2.63–2.76)
Low SDI	442.3 (161.4–737)	0.2 (0.1–0.3)	1,567.5 (614.9–2,540.4)	0.3 (0.1–0.5)	1.5 (1.38–1.62)	13,400.1 (4,984–22,374.7)	5.3 (1.9–8.8)	47,046.6 (18,740.2–75,740.4)	8.2 (3.2–13.2)	1.33 (1.22–1.45)
Region										
Europe										
Central Europe	3,627.9 (1,590.1–5,836.5)	2.5 (1.1–4)	6,940.6 (3,093–11,133.3)	3 (1.4–4.9)	0.56 (0.42–0.69)	87,450.4 (38,342.3–140,810.9)	57.8 (25.3–93)	148,738.2 (66,397.2–238,495.6)	68.9 (30.8–110.6)	0.48 (0.34–0.61)
Western Europe	12,481.1 (5,253.1–20,401.8)	2.1 (0.9–3.5)	18,025.5 (7,622.6–29,686.3)	1.8 (0.8–2.9)	−0.65 (−0.71–−0.59)	264,280.1 (111,569**–**431,654.1)	46.8 (19.7–76.5)	344,976 (147,806.6–564,627.8)	39.1 (16.8–63.5)	−0.67 (-0.74–−0.6)
Eastern Europe	5,381 (2,332–8,579.2)	1.9 (0.8–3.1)	9,051.6 (3,878.4–14,440.4)	2.5 (1.1–4)	0.72 (0.6–0.84)	137,909.2 (59,625–219,010.6)	48.6 (21–77.1)	208,723.8 (90,209.9–331,586.2)	60 (25.9–95.3)	0.45 (0.31–0.58)
America										
Andean Latin America	134.4 (54.9–221.2)	0.7 (0.3–1.1)	672.4 (287.4–1,130.6)	1.2 (0.5–1.9)	1.81 (1.69–1.94)	3,674.5 (1,506.5–5,995.5)	16.8 (6.9–27.4)	16,947.4 (7,443.7–28,548.2)	28 (12.3–47.2)	1.67 (1.55–1.79)
High-income North America	8,084.1 (3,450.1–13,011.4)	2.3 (1–3.6)	12,769.3 (5,673.4–19,869.6)	1.9 (0.9–3)	−0.66 (−0.76–−0.55)	185,196.9 (80,214.1–295,439.6)	54.5 (23.7–86.8)	296,819.6 (135,095.2–460,708.7)	49.5 (22.6–76.5)	−0.42 (−0.5–−0.33)
Southern Latin America	932.7 (403–1,520.7)	2.1 (0.9–3.4)	2,235.2 (992.4–3,641.5)	2.5 (1.1–4.1)	0.9 (0.73–1.08)	22,071.6 (9,492.6–36,141.3)	47.5 (20.4–77.6)	50,169.6 (22,345.7–81,467.6)	58.7 (26.1–95.2)	0.95 (0.8–1.11)
Central Latin America	529.6 (223.4–852.8)	0.7 (0.3–1.1)	3,148.9 (1,402.9–5,105.4)	1.3 (0.6–2.1)	2.11 (2.04–2.19)	14,473.4 (6,123.9–23,291)	16.1 (6.8–26)	84,536.9 (37,821.8–134,896.2)	32.8 (14.7–52.3)	2.32 (2.25–2.4)
Tropical Latin America	699.2 (289.8–1,135.9)	0.8 (0.3–1.3)	3,629.8 (1,533.5–5,850.4)	1.4 (0.6–2.3)	1.87 (1.76–1.98)	19,341.4 (8,091.7–31,333.8)	19.7 (8.2–32)	94,304.1 (39,800.4–150,657.5)	36 (15.2–57.6)	1.89 (1.79–2)
Asia										
Central Asia	488.2 (201.6–783.8)	1 (0.4–1.7)	768.9 (328.8–1,215.1)	1 (0.4–1.5)	0.1 (−0.03–0.22)	14,011.6 (5,760.4–22,453.1)	28.4 (11.7–45.4)	21,393.2 (9,092.7–33,879.8)	24.5 (10.4–38.7)	−0.22 (−0.32–−0.13)
East Asia	3,773 (1,309.3–6,405.9)	0.5 (0.2–0.8)	20,370.8 (8,474.7–33,798.9)	1 (0.4–1.6)	2.41 (2.34–2.48)	113,878.4 (39,419.4–193,735.9)	11.9 (4.1–20.3)	529,351.9 (219,132.7–886,681.3)	24.4 (10.1–40.8)	2.31 (2.21–2.41)
South Asia	516.9 (173.4–827.3)	0.1 (0–0.1)	3,042.7 (1,182.3–4,777.4)	0.2 (0.1–0.3)	2.81 (2.77–2.84)	16,693.8 (5,702.8–26,476.2)	2.5 (0.8–3.9)	91,500.2 (35,883–144,388.5)	5.7 (2.2–8.9)	2.74 (2.7–2.77)
Southeast Asia	652.6 (220.7–1,050.3)	0.2 (0.1–0.4)	4,057.3 (1,623–6,569.2)	0.6 (0.2–1)	3.04 (2.93–3.15)	20,952.7 (7,310.9–33,667.9)	7.1 (2.4–11.3)	119,340.4 (48,437.4–193,338.9)	16.6 (6.7–26.9)	2.84 (2.72–2.97)
North Africa and Middle East	1,341.1 (566–2,184.2)	0.8 (0.4–1.4)	5,636.8 (2,450.7–8,982.3)	1.3 (0.6–2.1)	1.7 (1.59–1.81)	39,567.7 (16,500.5–65,046.5)	21.2 (8.9–34.7)	156,482.3 (66,565–247,801.8)	31.8 (13.7–50.6)	1.46 (1.36–1.55)
High-income Asia Pacific	1,424.9 (521.3–2,304.2)	0.7 (0.3–1.2)	4,240 (1,633.5–6,743.7)	0.8 (0.3–1.3)	0.32 (0.27–0.36)	36,685.6 (13,456.3–59,450.7)	17.9 (6.6–29)	80,601.4 (31,642.1–127,988.1)	19.3 (7.6–30.5)	0.13 (0.09–0.18)
Africa										
Central Sub-Saharan Africa	50.4 (18.9–86.2)	0.2 (0.1–0.4)	254.1 (96.4–437.8)	0.5 (0.2–0.8)	2.41 (2.24–2.57)	1,494.1 (554.6–2,559.9)	6 (2.2–10.1)	7,638.1 (2,874.6–13,167.7)	12.2 (4.6–21)	2.34 (2.19–2.5)
Eastern Sub-Saharan Africa	208.5 (73.4–349.5)	0.3 (0.1–0.5)	773.8 (295.2–1,284.4)	0.5 (0.2–0.8)	1.62 (1.52–1.72)	6,358.8 (2,271.4–10,621.8)	7.5 (2.7–12.6)	23,026.2 (8,703.5–37,649)	12 (4.6–19.8)	1.33 (1.23–1.43)
Southern Sub-Saharan Africa	197.2 (83.5–310)	0.8 (0.3–1.2)	807.9 (341.4–1,269.9)	1.5 (0.6–2.3)	2.32 (2.06–2.58)	5,488.2 (2,315.6–8,602.3)	18.8 (8–29.5)	21,915.4 (9,192.3–34,323)	35.7 (15–55.8)	2.36 (2.1–2.62)
Western Sub-Saharan Africa	193.8 (76.5–312.9)	0.2 (0.1–0.4)	834 (336.3–1,365.1)	0.5 (0.2–0.8)	2.39 (2.33–2.45)	5,310.9 (2,112.9–8,558.9)	5.7 (2.3–9.2)	22,886 (9,127.6–37,858.8)	10.8 (4.3–17.7)	2.2 (2.14–2.25)
Caribbean	243.8 (101.8–387.5)	1 (0.4–1.5)	841.4 (355.2–1,385)	1.6 (0.7–2.6)	1.68 (1.62–1.73)	6,308.4 (2,656.5–10,111.5)	23.8 (10–38.2)	20,386.2 (8,687.1–33,782.8)	37.9 (16.2–62.9)	1.66 (1.61–1.71)
***Oceania***						470.6 (193.9–787.7)	13.6 (5.5–22.8)	1,362 (562.2–2,191.1)	15.5 (6.4–25.1)	0.44 (0.37–0.52)
Australasia	560.6 (233.2–893.9)	2.4 (1–3.8)	1,123.4 (476.3–1,796.5)	2 (0.9–3.2)	−0.78 (−0.86–−0.71)	13,424.1 (5,579.5–21,236)	58.2 (24.2–91.9)	23,565.4 (10,170–37,480.5)	46.7 (20.2–74.4)	−0.94 (−1.02–−0.85)
Oceania	14.7 (5.9–24.5)	0.5 (0.2–0.8)	43.5 (18–70.3)	0.6 (0.2–1)	0.51 (0.42–0.6)	470.6 (193.9–787.7)	13.6 (5.5–22.8)	1,362 (562.2–2,191.1)	15.5 (6.4–25.1)	0.44 (0.37–0.52)

ASDR, age-standardized death rate; DALYs, Disability-adjusted life-years.

### Patterns across SDI quintiles

Disease burden varied significantly among the different SDI quintiles. High SDI regions have shown a consistent reduction in ASDR in recent years (EAPC_ASDR_ = −0.64, 95%UI: −0.69 to −0.59; EAPC_DALYs_ = −0.48, 95%UI: −0.52 to −0.43). Despite this progress, these regions still carried the highest global burden in 2021, with 36,529.9 (95% UI 15,670.3–58,138.7) deaths, 1.7 (95% UI 0.7–2.7) ASDR and 775,808.6 (95% UI 337,834.3–1,225,933.9) DALYs (**Supplementary Table 1**, **Table 1**, **Figures 1A–F**). Following closely behind were the high-middle and middle SDI regions, with middle SDI regions standing out because of a striking increase in burden. Specifically, the middle SDI regions exhibited dramatic increases in burden, with an ASDR EAPC of 2.13 (95% CI 2.11–2.16) and a DALY rate EAPC of 2.1 (95% CI 2.07–2.14). On the other hand, low-middle and low SDI regions, while reporting the lowest absolute burden, displayed worrisome upward trends (EAPC_ASDR_ = 2.72, 95%UI 2.65–2.79; EAPC_DALYs_ = 2.692.69, 95%UI 2.63–2.76) (**Supplementary Table 1**, **Table 1**, **Figures 1A–F**). Subsequently, we used the frontier analysis to examine the correlation between DALYs and a country’s developmental status. With an increase in the SDI, the effective difference tends to become less prominent. The countries exhibiting the largest effective difference from the frontier included Poland, Greenland, Romania, and Czechia. However, a discernible divergence from the frontier was observed in Denmark, Canada, and the Netherlands, indicating the potential for further refinement in health outcomes despite their advanced developmental status (**Figure 1J**). In addition, absolute inequality, as measured by the slope index, increased substantially from 1990 to 2021, reflecting a widening gap in the CRC burden between countries ranked by SDI. Moreover, the total number of CRC cases has shown a consistent upward trajectory for both sexes over the past three decades (**Figure 1K**). This increase is expected to continue, with the total number of cases for both sexes projected to surpass 150,000 annually by 2040 (**Figure 1L**).

### Regional burden of colorectal cancer attributable to high BMI

In 2021, East Asia had the highest absolute mortality burden of CRC attributable to high BMI, with 20,370.8 (95% UI 8,477.4–33,798.9) deaths, accounting for over 20% of global deaths (**Supplementary Table 1**, **Figures 1M, N**). East Asia also reported one of the highest DALY counts, with 529351.9 (95% UI 219132.7–886681.3) (**Table 1**, **Figures 1M, N**). These numbers were accompanied by an ASDR of 1 (95% UI 0.4–1.6) and an age-standardized DALY rate of 24.4 (95% UI 10.1–40.8), both of which showed positive EAPCs of 2.41 (95% CI 2.34–2.48) and 2.31 (95% CI 2.21–2.41), respectively. Additionally, Western Europe exhibited the second highest disease burden, with 18,025.5 deaths (95% UI 7,622.6–29,686.3) and 1.8 ASDR (95% UI 0.8–2.9); while demonstrating a decreasing trend (EAPC_ASDR_: −0.65, 95% UI: −0.71 to −0.59) (**Supplementary Table 1**, **Figures 1M, N**). The burden of CRC mortality was also considerable in Central and Eastern Europe, with 148,738.2 (95% UI 66,397.2–238,495.6) and 208,723.8 (95% UI 90,209.9–331,586.2) DALYs reported in 2021, respectively (**Table 1**, **Figures 1M, N**). The Americas has also demonstrated marked disparities in CRC mortality and DALYs across regions. High-income North America, including the United States and Canada, had the third highest disease burden, reporting 12,769.3 (95% UI 5,673.4–19,869.6) deaths, 1.9 ASDR (95% UI 0.9–3.0), and 296819.6 DALYs (95% UI 135095.2–460708.7). Similar to Western Europe, the trend in North America showed a slight decline (EAPC_DALYs_ = −0.42, 95%UI −0.5 to −0.33) (**Supplementary Table 1**, **Table 1**). In contrast, Australasia showed the lowest ASDR and DALY rates, at 0.2 (95% UI 0.1–0.3) and 5.5 (95% UI 4.2–9.0), respectively. Notably, Australasia also exhibited a declining trend over time, with an EAPC of −0.78 (95% CI −0.86 to −0.71) for ASDR and −0.94 (95% CI −1.02 to −0.85) for DALYs (**Supplementary Table 1**, **Table 1**).

### Burden of lung cancer attributable to particulate matter pollution by countries

China, the USA, Russia, Brazil, and Germany consistently ranked among the highest for both all-age mortality counts and DALYs attributable to high BMI-related CRC in 2021 (**Figures 1M, N**; **Supplementary Table 1**, **Table 1**). Notably, China reported the highest figures with 19,417.6 mortality cases and 507,316.5 DALYs, followed by the USA with 11,401.7 deaths and 268,295.7 DALYs. Despite their high disease burden, the trends in ASDRs and DALYs showed differences across these countries. China reported an EAPC of 2.39 (95% CI 2.31–2.48) for ASDRs and 2.31 (95% CI 2.19–2.42) for DALYs, indicating a persistent increase. However, the USA showed marginal declines in disease burden, with an EAPC of −0.69 (95% CI −0.8 to −0.57) for ASDR and −0.41 (95% CI −0.5 to −0.32) for DALYs. Russia exhibited a stable trend, with an EAPC of 1.05 (95% CI 0.88–1.22) for ASDR and 0.72 (95% CI 0.54–0.9) for DALYs. Brazil, on the other hand, experienced a notable increase, with an EAPC of 1.84 (95% CI 1.73–1.95) for ASDR and 1.87 (95% CI 1.76–1.97) for DALYs. Germany reported a marginal decrease in ASDR (EAPC: −1.74, 95% CI −1.86 to −1.62) and in DALYs (EAPC: −1.65, 95% CI −1.77 to −1.54). In addition, Hungary (ASDR: 3.8, 95% UI: 1.7-6.2), Slovakia (3.5, 95% UI: 1.6-5.7), Uruguay (3.5, 95% UI: 1.5–5.7), Bulgaria (3.4, 95% UI: 1.4–5.6), and Croatia (3.4, 95% UI: 1.5–5.4) recorded the highest ASDRs in 2021.

## Discussion

To our knowledge, this is the first comprehensive analysis of the global burden of CRC attributable to high BMI, revealing profound implications for public health and global equity. Our study emphasizes several important patterns. First, over the past 30 years, the CRC burden has more than doubled, and the projected increase in CRC cases may be over 150,000 annually by 2040. Moreover, the disease burden was disproportionately higher in males than in females. Second, high-income regions such as Western Europe and North America, while experiencing a decline in age-standardized death and disability rates due to high BMI-related CRC, still carry a disproportionately large share of the global burden. This paradox reflects both the lingering consequences of historical lifestyle patterns and the challenges of managing CRC in aging populations (10). Third, high-middle- or middle-income regions, especially China, Russia, and Brazil, are witnessing a dramatic surge in the burden of CRC. Although East Asia has undergone rapid economic development and urbanization in recent decades, these advances have been accompanied by significant shifts in dietary patterns, physical activity, and lifestyle behaviors, contributing to the increasing prevalence of obesity and related diseases, including CRC (11, 12). This upward trajectory is particularly concerning, given the strain it places on healthcare systems that may lack the capacity to manage such a complex disease effectively. Fourth, low middle- or low-income regions, while reporting the lowest absolute burden, showed worrisome upward trends in CRC incidence and mortality. These increases may be attributed to the double burden of malnutrition and obesity as well as limited access to early detection and treatment. Moreover, there was a substantial increase in absolute inequality in the burden between the high and low SDI regions. The relationship between the increasing CRC burden and high BMI represents a global challenge with far-reaching implications. Addressing this issue requires multifaceted approaches that include the widespread promotion of healthy lifestyles, implementation of effective weight management programs, and greater emphasis on primary prevention through dietary and physical activity interventions. Furthermore, strengthening health systems to provide equitable access to CRC screening and treatment remains critical in mitigating the burden of the disease, particularly in low- and middle-income countries.

## Conclusion

This study highlights the significant and rising global burden of CRC attributable to high BMI, with disparities across SDI regions. While high-SDI regions showed declining rates, they retained a substantial burden, whereas low- and middle-SDI regions faced alarming upward trends. Urgent, multifaceted interventions targeting prevention, early detection, and equitable healthcare access are essential for mitigating the growing CRC burden and reducing premature mortality from non-communicable diseases.

## Data Availability

The raw data supporting the conclusions of this article will be made available by the authors, without undue reservation.
